# Long-term toxicity of ZnO nanoparticles to *Scenedesmus rubescens* cultivated in different media

**DOI:** 10.1038/s41598-017-13517-7

**Published:** 2017-10-18

**Authors:** Andriana F. Aravantinou, Fytoula Andreou, Ioannis D. Manariotis

**Affiliations:** Environmental Engineering Laboratory, Civil Engineering Department, University of Patras, Patras, 26504 Greece

## Abstract

The aim of this work was to investigate the long-term toxic effect of zinc oxide (ZnO) nanoparticles (NPs) on freshwater microalgae, combined with the nutrient consumption in the culture. For this purpose, two common microalgae media (Blue-Green 11, BG-11, and Bold’s Basal Medium, BBM) were used. *Scenedesmus rubescens* was used as freshwater microalgae model species and was exposed to ZnO NPs at different concentrations (0.081 to 810 mg/L) for a period up to 28 d. The experimental results revealed that microalgae growth was affected by the time of exposure and the NPs concentrations, but mainly the culture medium used. Differences in microalgae growth rates were observed and attributed to the selected culture medium. The toxic effect of ZnO NPs was higher on microalgae cultured in modified BG-11 compared to BBM, despite the fact that *S. rubescens* exhibited higher growth rate in modified BG-11 without the exposure of ZnO NPs.

## Introduction

Nanoparticles (NPs) have always existed in the natural environment (soil, water and atmosphere)^1^. Although exposure to NPs is not a new phenomenon, the rapid development of commercial applications, involving the use of a large variety of engineering NPs, has resulted in the introduction of quite higher amounts into the environment^2^. Engineering NPs have been found in wastewater, which are directed to treatment plants, and then discharged, in the aquatic environment^3,4^. As the use of NPs increases, their effect to the coastal food chain and ecosystems is crucial. It should be mentioned that the legislative framework concerning the monitoring and the potential harmful effects of NPs is limited^5^.

Nanoparticles of metal oxides, such as CuO, ZnO, TiO_2_, Ag, CeO_2_, and SiO_2_ have been observed to inhibit the growth of various algae^6,7^. Among all these metal oxide NPs, ZnO NPs are noted for their chemical stability and strong adsorption ability and have been extensively used in commercial products like sunscreens, coatings, and paints^8^. ZnO NPs can be found in surface waters at high concentrations, posing significant threat to aquatic ecosystems^7,9^. Previous studies have demonstrated that ZnO NPs are toxic to microorganisms, cells, plants, aquatic biota and rodents^8,10,11^. Aruoja *et al*.^12^ investigated the effect CuO, ZnO and TiO_2_ NPs to *Pseudokirchneriella subcapitata*. The most toxic metal oxide to algae was ZnO, and the toxicity of ZnO NPs was attributed to the soluble metal ions derived from nanoparticles. ZnO NPs are toxic not only to microalgae, but also to other aquatic microorganisms. Zhu *et al*.^13^ found that ZnO NPs exhibited higher acute toxicity to zebrafish embryos than TiO_2_ and Al_2_O_3_ NPs.

Recent studies^8,9,12–17^ have shown that ZnO NPs have toxic effects on microalgae, which depend on the species type, exposure time, NPs concentration, and mainly the culture medium. Few studies have focused on NPs behavior-mechanisms in different environmental conditions, and other in the effect-mechanisms on different organisms. The species type of microalgae (freshwater or marine) define the behavior of NPs and the toxic effects mechanisms since NPs dissolution depend on the aqueous matrix composition (pH, ionic strength, organic matter content, etc.). Manzo *et al*.^16^ reported that ZnO NPs were more toxic for the marine algal *Dunaliella tertiolecta* than bulk ZnO, while the toxic effects could not be strictly related to the action of zinc metal ion. Aravantinou *et al*.^11^ investigated the effect of ZnO NPs on freshwater (*Chlorococcum* sp. and *Scenedesmus rubescens*) and marine (*Dunaliella tertiolecta* and *Tetraselmis suesica*) microalgae species. They reported that the sensitivity of algae to ZnO NPs strongly depended on the species type, the concentration of NPs and the medium composition. Specifically, *S. rubescens* presented higher viability compared to the other species, but its inhibition was greater when it was cultured in BBM compared to 1/3N BG-11 medium (BG-11 enriched with one third times the nitrates).

In high concentrations NPs, aggregates and may wrap on the microalgae cell, while at lower concentrations particle size decrease^11,18^ and toxicity increase^18^. The formation of aggregations does not mean low toxicity^19^, even though they reduce NPs dissolution^6^. The dissolved Zn ions were not the dominant mechanism for the *Chlorella sp*. growth inhibition^6^. The Zn ions were more toxic than ZnO NPs for zinc concentrations lower than 50 mg/L, while ZnO NPs exhibited higher toxicity than Zn ions at concentrations greater than 50 mg/L^6^.

The culture medium is the main factor for the ZnO NPs behavior and microalgae growth. The BG-11 and BBM culture media are commonly used for freshwater algae^20^, especially for *S. rubescens*. The two media have different zinc concentration, which explain the difference in the toxic effect of ZnO NPs and the Zn ions on microalgae^11^. The above studies have substantially increased our knowledge of the toxic effects of metal oxides NPs, based on short-term toxicity tests. However, more studies are clearly needed to clarify both the toxicological effects and underlying mechanism of nanoparticles in long-term exposure, which resembles the environmental conditions.

The aim of this work was to investigate the long-term toxic effects and the effect on the metabolic reactions of ZnO NPs on freshwater microalgae, cultured in different mediums. *S. rubescens* was used as freshwater microalgae model species, since in previous studies^11^ it was observed to change with time, becoming more resistant after 4 days of exposure to ZnO NPs. The effect on the metabolic reactions of microalgae, in the presence of NPs, was determined by monitoring the growth rate, the nutrient removal and lipids production of microalgae during exposure to different concentrations of NPs. According to our knowledge no previous study has investigated the exposure of microalgae on ZnO NPs for a long-term period.

## Results and Discussion

### ZnO NPs effects on microalgae cultures

**Figure 1:**
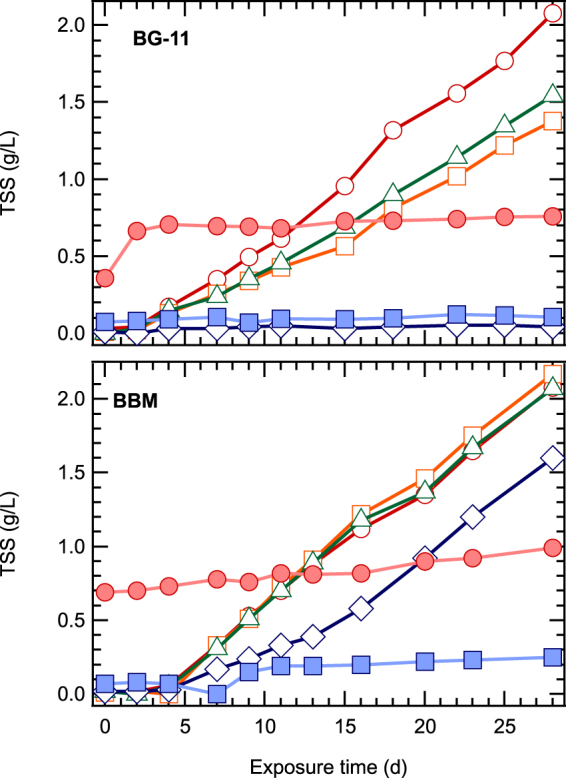
Effect of different ZnO NPs concentrations (○ 0, □ 0.081, Δ 0.81, ◊ 8.1, ■ 81, ● 810 mg/L ZnO NPs) on biomass of *S. rubescens* cultured in 1/3N BG-11 and BBM media.

**Figure 2:**
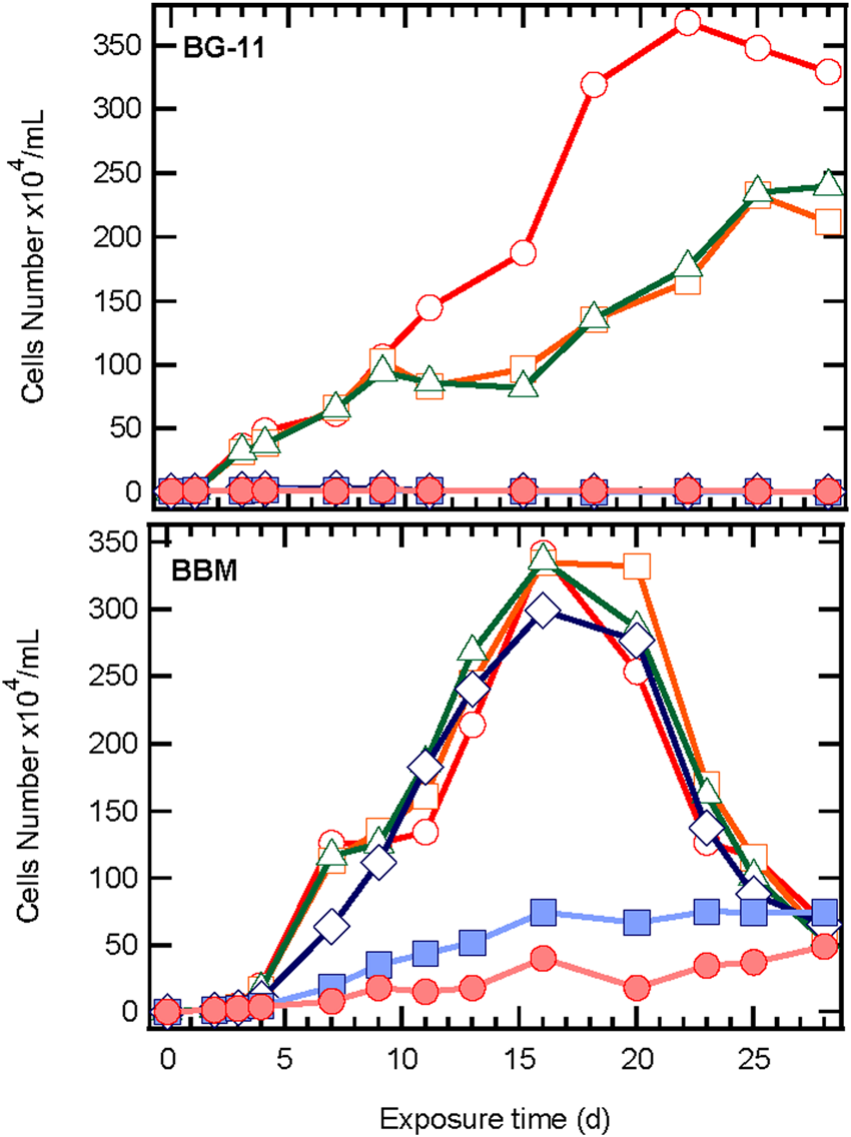
Effect of different ZnO NPs concentrations (○ 0, □ 0.081, Δ 0.81, ◊ 8.1, ■ 81, ● 810 mg/L ZnO NPs) on cell numbers of *S. rubescens*, cultured in 1/3N BG-11 and BBM media.

**Figure 3:**
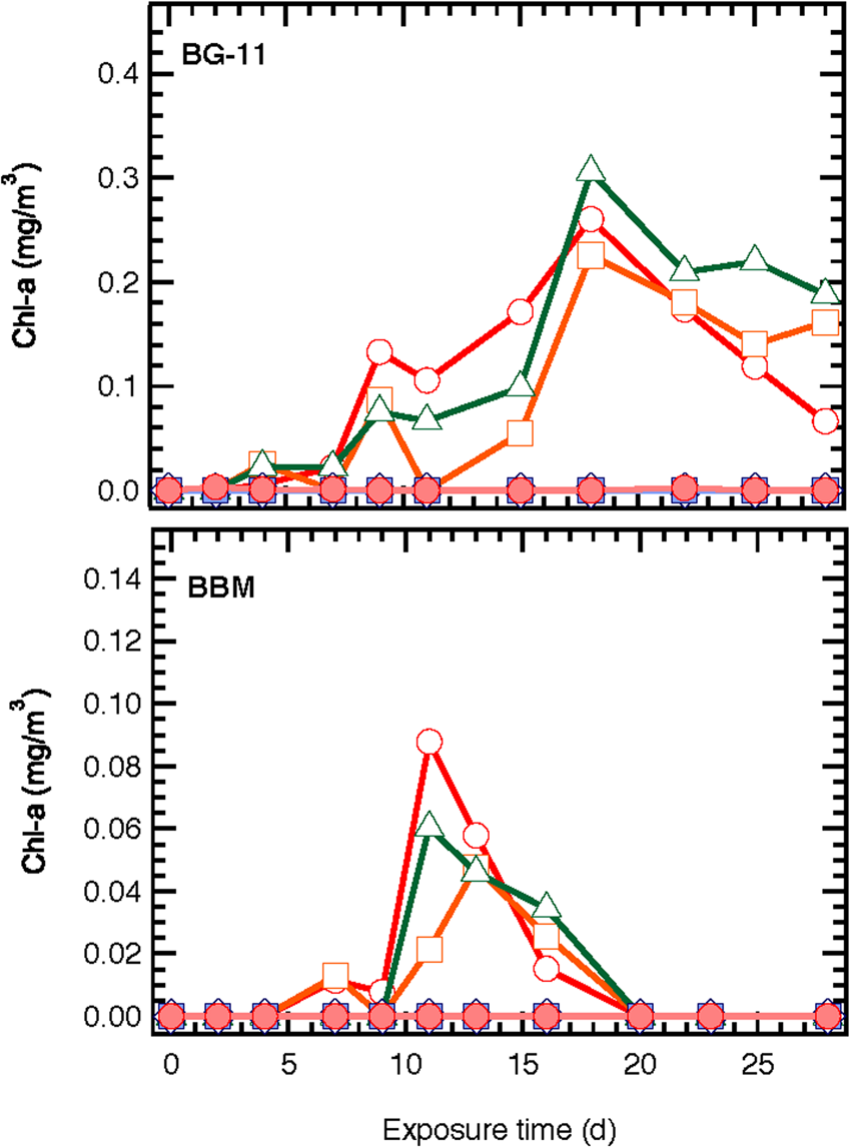
Effect of different ZnO NPs concentrations (○ 0, □ 0.081, Δ 0.81, ◊ 8.1, ■ 81, ● 810 mg/L ZnO NPs) on chl-a concentration of *S. rubescens* cultured in 1/3N BG-11 and BBM media.

Microalgae biomass concentrations are presented in Fig. 1. ZnO NPs at low concentrations did not affect the growth of microalgae, in contrast to higher levels (≥8.1 mg/L) where microalgae growth was almost zero. At 810 mg/L ZnO NPs, the biomass concentration remained constant at 0.75 g/L after the third day, which was attributed to the concentration of NPs added in the culture (Fig. 1a). This implies that after two days NPs formed agglomerations, which were completely retained in the filter, while in the first day a fraction of 57% of NPs passed through the filter. Similar results were reported by Hartmann *et al*.^21^, who studied different types of TiO_2_ NPs and observed that after 72 h larger sized aggregates were formed. The results of *S. rubescens cultures* in the BBM medium (Fig. 1b) presented similar growth in tests with 0.081 and 0.81 mg/L ZnO NPs, and similar behavior with the culture in 1/3N BG-11 medium. Moreover, it was observed that the microalgae culture in BBM exhibited tolerance to higher NPs concentrations than culturing in 1/3N BG-11, especially at 8.1 mg/L ZnO NPs. However, for NPs concentrations higher than 81 mg/L ZnO NPs, the growth of microalgae was almost zero. The biomass concentration was estimated more precisely via the cell numbers (Fig. 2) and chlorophyll-a (chl-a) (Fig. 3), since the ZnO NPs formed agglomerations when added to the media.

Figure 2 shows the results of cell numbers cultured in 1/3N BG-11 and BBM media in the presence or absence of ZnO NPs. The first plot shows cultures of *S. rubescens* in 1/3N BG-11 medium, and in this case the growth of microalgae was higher in the absence of ZnO NPs than in cultures containing 0.081 and 0.81 mg/L ZnO NPs. This implies toxic impact of ZnO NPs on microalgae growth. Specifically, in the absence of ZnO NPs the cells number reached up to 368 × 10^4^ cells/mL after 22 days of operation compared to the cultures containing 0.081 and 0.81 mg/L ZnO NPs, which had maximum numbers of 233 and 235 × 10^4^ cells/mL, respectively on the 25^th^ day. The cultures in BBM medium reached the highest cell number in shorter time compared to 1/3N BG-11 medium cultures. In the presence of ZnO NPs at concentrations ≤ of 8.1 mg/L, algal growth was sustained until the 16^th^ day and then a gradual decline of cells number was observed. Microalgae cultured in BBM medium were more resistant in the presence of higher ZnO NPs concentrations compared to cultures in BG-11 medium.

The maximum chl-a concentration, in the control culture with 1/3N BG-11 (Fig. 3), was 0.31 mg/L and was observed on the 18^th^ day. On the same day the chl-a concentration in the cultures with 0.081 and 0.81 mg/L ZnO NPs were 0.22 and 0.25 mg/L, respectively. In the BBM cultures the maximum chl-a values of 0.09 and 0.06 mg/L were observed in the control and 0.81 mg/L ZnO NPs on the 11^th^ day, while a value of 0.05 mg/L was determined on the 13^th^ day on the 0.081 mg/L ZnO NPs culture. The chl-a concentration was zero in cultures with NPs concentration ≥8.1 mg/L for both media. The low chl-a concentrations may be explained by the properties of NPs, which could wrap up the algal cells leading to shading^22^, or could enter the algal cells and attach on the mitochondria^4,23,24^ resulting in the reduction of cell photosynthesis.

**Figure 4:**
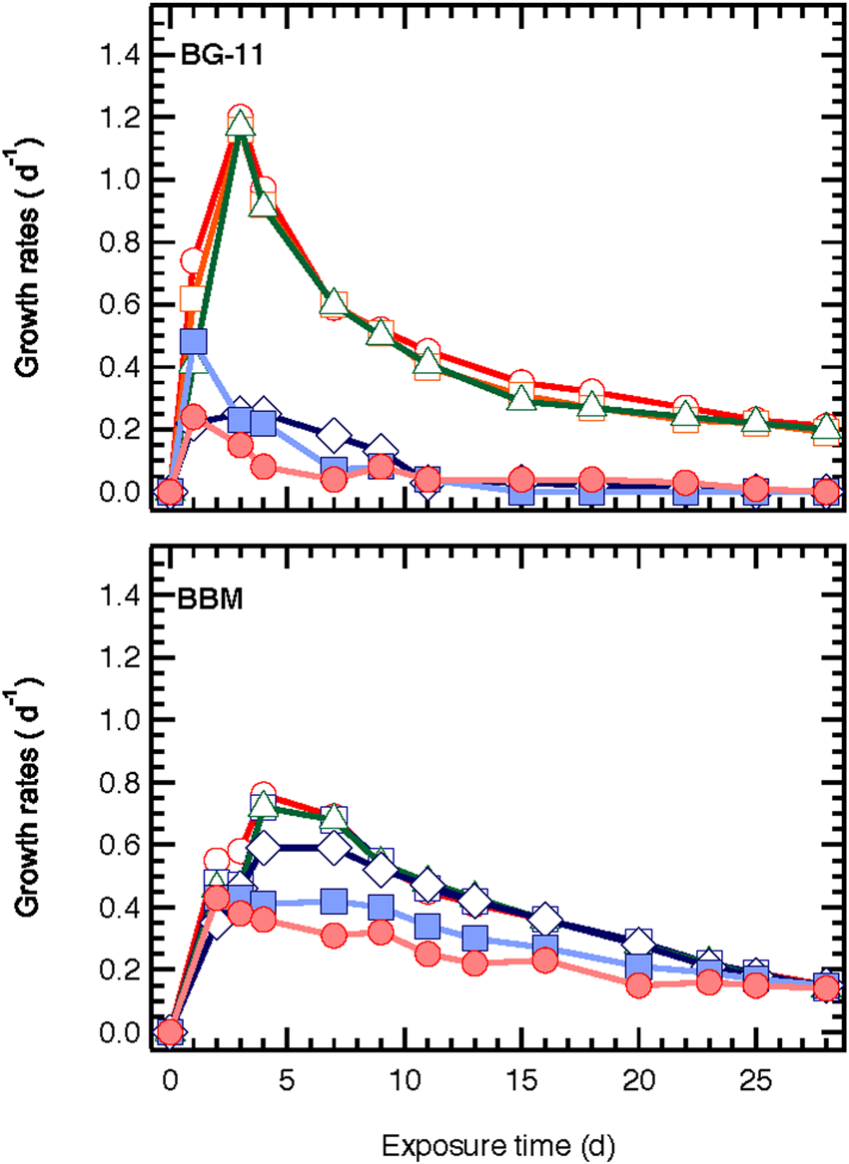
Growth rates of *S. rubescens* cultures after exposure to different ZnO NPs concentrations (○ 0, □ 0.081, Δ 0.81, ◊ 8.1, ■ 81, ● 810 mg/L ZnO NPs) and cultured in 1/3N BG-11 and BBM media.

The toxic effect of ZnO NPs on *S. rubescens* was higher in 1/3N BG-11 than in BBM cultures, despite the fact that *S. rubescens* exhibited higher growth rate in 1/3N BG-11 when it was not exposed on ZnO NPs (Fig. 4). Specifically, in the presence of 8.1 mg/L ZnO NPs cultures demonstrated higher growth rates in the BBM than the 1/3N BG-11 medium.

### ZnO NPs- induced effects on algal nutrients uptake

**Figure 5:**
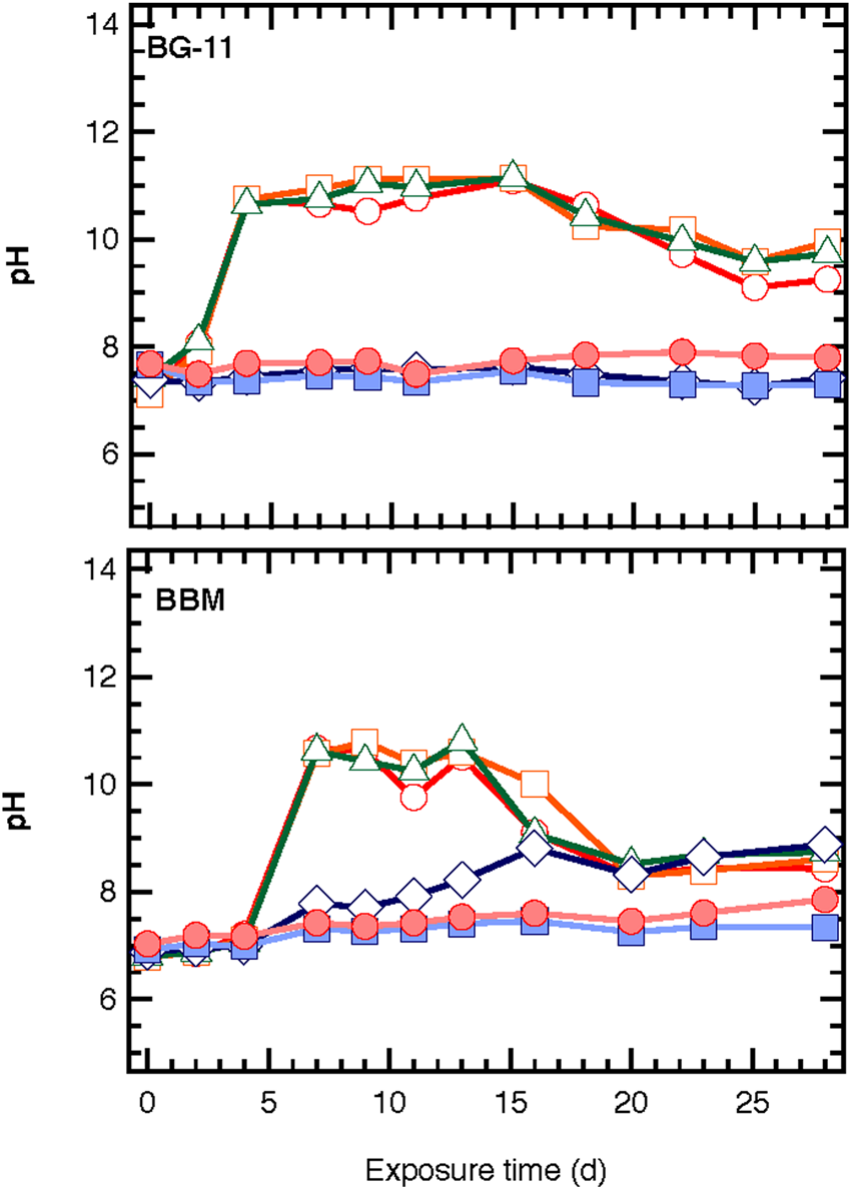
pH values of *S. rubescens* cultures after exposure to different ZnO NPs concentrations (○ 0, □ 0.081, Δ 0.81, ◊ 8.1, ■ 81, ● 810 mg/L ZnO NPs) and cultured in 1/3N BG-11 and BBM media.

The pH values ranged from 7 to 12 and 6 to 11, in 1/3N BG-11 and BBM media cultures, respectively (Fig. 5). Changes in pH were observed in the control and the 0.081 and 0.81 mg/L ZnO NPs cultures, which showed increases in algal biomass. These changes were anticipated because of the metabolic activities of microalgae cells, which increase the pH of the solution in the culture^19^. A smaller increase in pH occurred in BBM cultures at 8.1 mg/L ZnO NPs. pH values were similar in 1/3N BG-11 cultures containing 0 to 0.81 mg/L ZnO NPs, and were much higher with 8.1 mg/L. A plateau was observed between 4 and 15 d followed by a drop of pH values.

**Figure 6:**
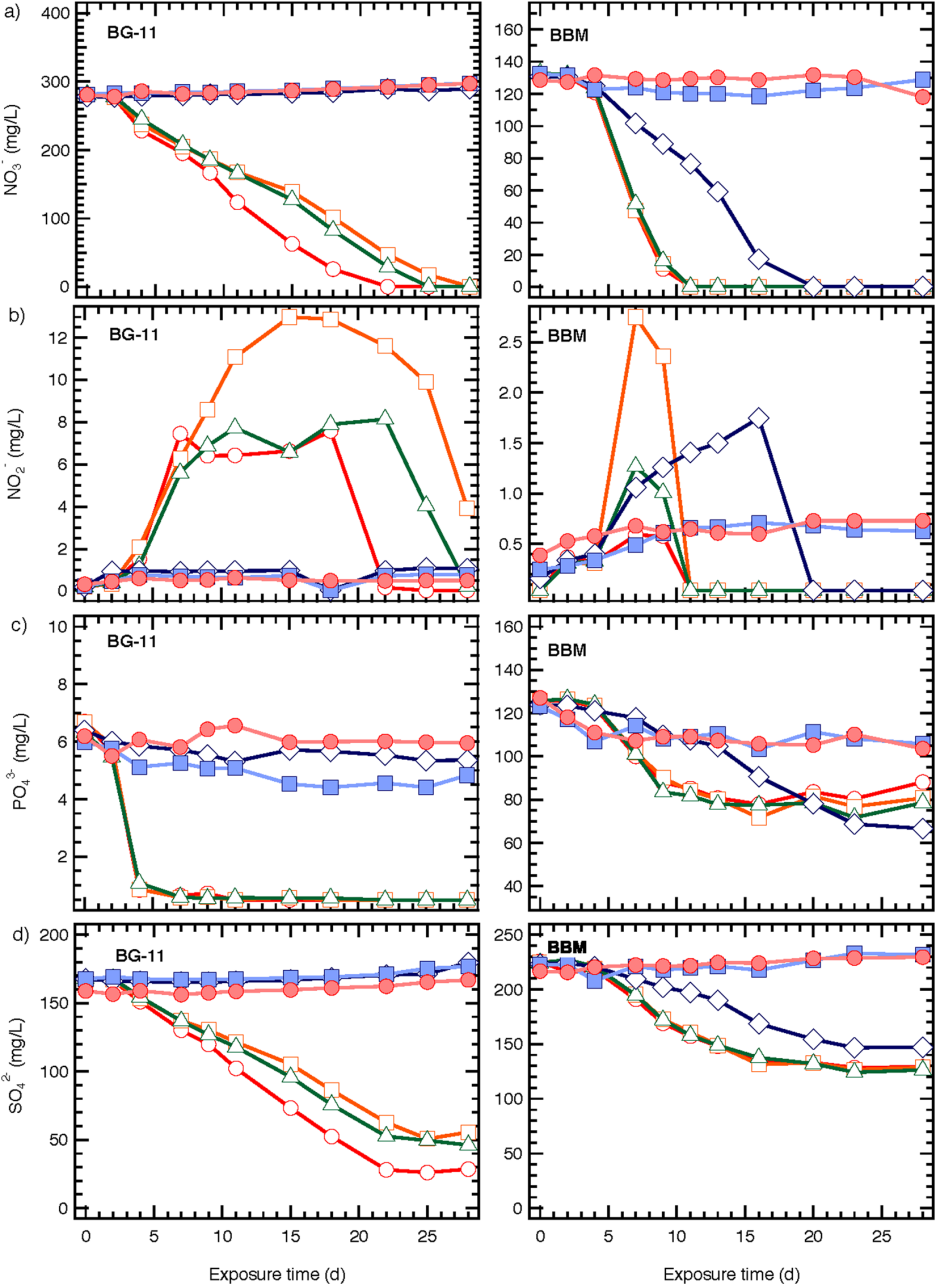
Nutrients concentration of *S. rubescens* cultures after exposure to different ZnO NPs concentrations (○ 0, □ 0.081, Δ 0.81, ◊ 8.1, ■ 81, ● 810 mg/L ZnO NPs) and cultured in 1/3N BG-11 and BBM medium.

Figure 6a and b show nutrient removal in the cultures during the exposure to ZnO NPs. Nitrate (Fig. 6a) and nitrite (Fig. 6b) data indicate that the reduction of nitrates ensures the increase of nitrites in both culture media (the media did not contain nitrites). This increase was observed in cultures in both media and at ZnO NPs from 0 (control culture) to 0.81 mg/L, which exhibited microalgae biomass growth, and occurred at the beginning of exposure. In the next days a gradual plateau was attained in the 1/3N BG-11 cultures for concentrations up to 0.81 mg/L. At higher NPs levels nitrites were close to zero. Cultures in BBM medium had quite lower nitrites concentration, and this was attributed to the lower content of nitrates in the medium, compared to 1/3N BG-11 and their rapid depletion. Figure 6a shows the gradual reduction of nitrates to zero in the algal control cultures and those with low NPs levels (≤0.81 mg/L with 1/3N BG-11 and ≤8.1 mg/L with BBM medium). In cultures with high NPs content the nitrate level remained constant, since there was no any algal growth. The nitrate and nitrite data imply the effects on microalgae metabolism after exposure on ZnO NPs. Specifically, in cultures with BBM medium and in presence of 8.1 mg/L ZnO NPs the nitrates uptake from microalgae cells was not at the same rate as in the control cultures, despite the fact that they demonstrated similar cell numbers. At day 11 the algal yield per nitrates consumed was 9 × 10^6^ and 2 × 10^7^ cells/mg NO_3_^−^ in the control and 8.1 mg/L ZnO NPs culture, respectively. This would suggest that the presence of NPs could cause dysfunctions in algal metabolic activities. Moreover, the nitrites concentrations increased after the 4^th^ day of cultivation while the algal growth rates (Fig. 4) were decreased, and possibly lysis of the dead algae cells occurred. Due to cell lysis, nitrites were released into the environment resulting in the increased concentrations. In cultures exposed to 0.081 to 8.1 mg/L ZnO NPs high nitrite levels were observed compared to control culture, although they demonstrated similar cell numbers. This implies that cell lysis was increased in the presence of NPs.

Phosphates were completely removed in 1/3N BG-11 cultures from the first days of operation even in the presence of low concentrations of ZnO NPs (≤0.81 mg/L) (Fig. 6c). Phosphates are essential nutrients for algal growth, as nitrates are. BBM cultures containing less than 8.1 mg/L ZnO NPs exhibited decrease in phosphorous concentration, while at higher NPs levels removal was negligible. The decrease in phosphates concentration followed that of nitrates for NPs concentrations up to 8.1 mg/L, and there was no additional reduction after nitrates were completely removed (10^th^ day). This indicated that consumption of phosphates and nitrates was in synergy. A significant difference was observed in phosphates consumption in both media at NPs concentration of 8.1 mg/L. At a NPs concentration of 810 mg/L phosphates reduction was observed in both media cultures, which was attributed to binding of phosphates on NPs agglomerations. Ji *et al*.^6^, reported that nutrients such as phosphorus might be adsorbed on NPs (TiO_2_), enhancing their removal from the algal growth medium. In the case of BG-11 medium complete removal of phosphates and nitrates was observed at the 28^th^ day of operation for NPs levels up to 8.1 mg/L, despite the initial high concentration of the nutrients at the beginning of the experiments. Sulphate removal was also observed and was due to algal growth. In 1/3N BG-11 cultures removal was observed in the presence of NPs up to 0.81 mg/L, while in BBM cultures sulphates removed more rapidly at NPs levels up to 8.1 mg/L.

In summary, at first glance, nutrient removal was analogous to algal biomass growth. In the presence of the highest ZnO NPs concentrations (81 and 810 mg/L), inhibition of biomass growth was up to 100% and nutrients did not decrease during the operation period. The presence of ZnO NPs affects the nutrient uptake by microalgae, even at low concentrations, with unpredictable long-term effects, as the impact on microalgae metabolic activities is not clear yet.

### ZnO NPs- induced effects on algal lipid accumulation

**Figure 7:**
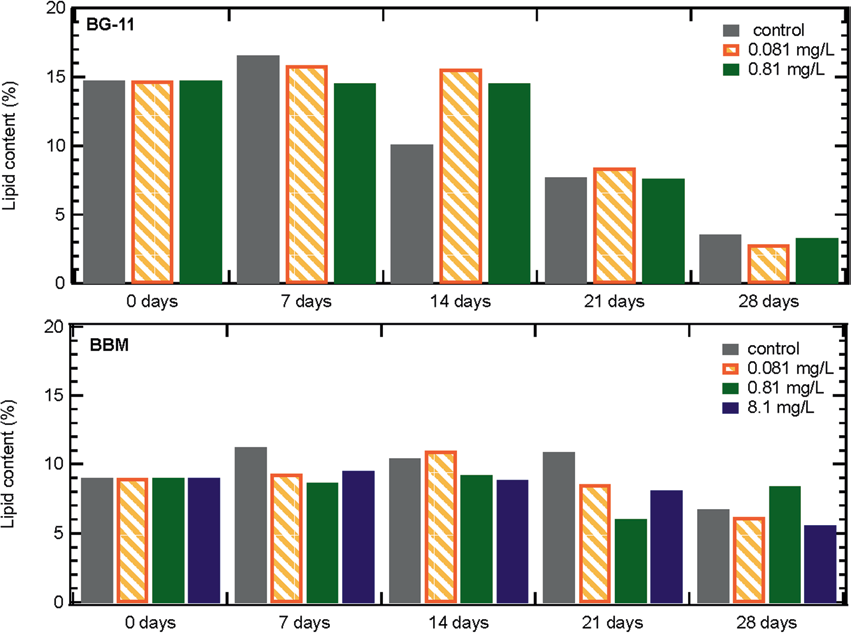
Effect of different ZnO NPs concentrations on the lipid content of *S. rubescens* cultured in 1/3N BG-11 and BBM medium.

The lipid content of algal biomass is presented in Fig. 7. *S. rubescens* cultured in 1/3N BG-11 had the highest lipid content value on the 7^th^ day of operation for ZnO NPs concentration from 0 to 0.81 mg/L. In the absence of NPs, the algal lipid content was immediately reduced, which was not the case in cultures exposed to ZnO NPs at 0.081 and 0.81 mg/L. In the presence of NPs, the lipid content on the 14^th^ day was similar to the 7^th^ day value, while a significant decrease occurred after the 21^st^ day. *S. rubescens* cultured in BBM presented lower lipid content than in cells cultured in 1/3N BG-11 medium, although, in BBM medium it showed lower biomass inhibition in the presence of ZnO NPs. Specifically, in the presence of 8.1 mg/L ZnO NPs, *S. rubescens* in BBM showed the ability to increase its biomass and lipid content. The latter was not observed in 1/3N BG-11 medium cultures. Finally, for the cultures without and with 0.081 mg/L ZnO NPs, lipid content reached up to 15 and 10%, respectively for 1/3N BG-11and BBM media. The high lipid content in the presence of low ZnO NPs concentration is due to oxidative stress.

The exposure to NPs and lack of nutrients can cause oxidative stress on the cells^11^, which leads to the generation of reactive oxygen species (ROS) and to lipid accumulation^25^.

### Evaluation of toxicity

**Figure 8:**
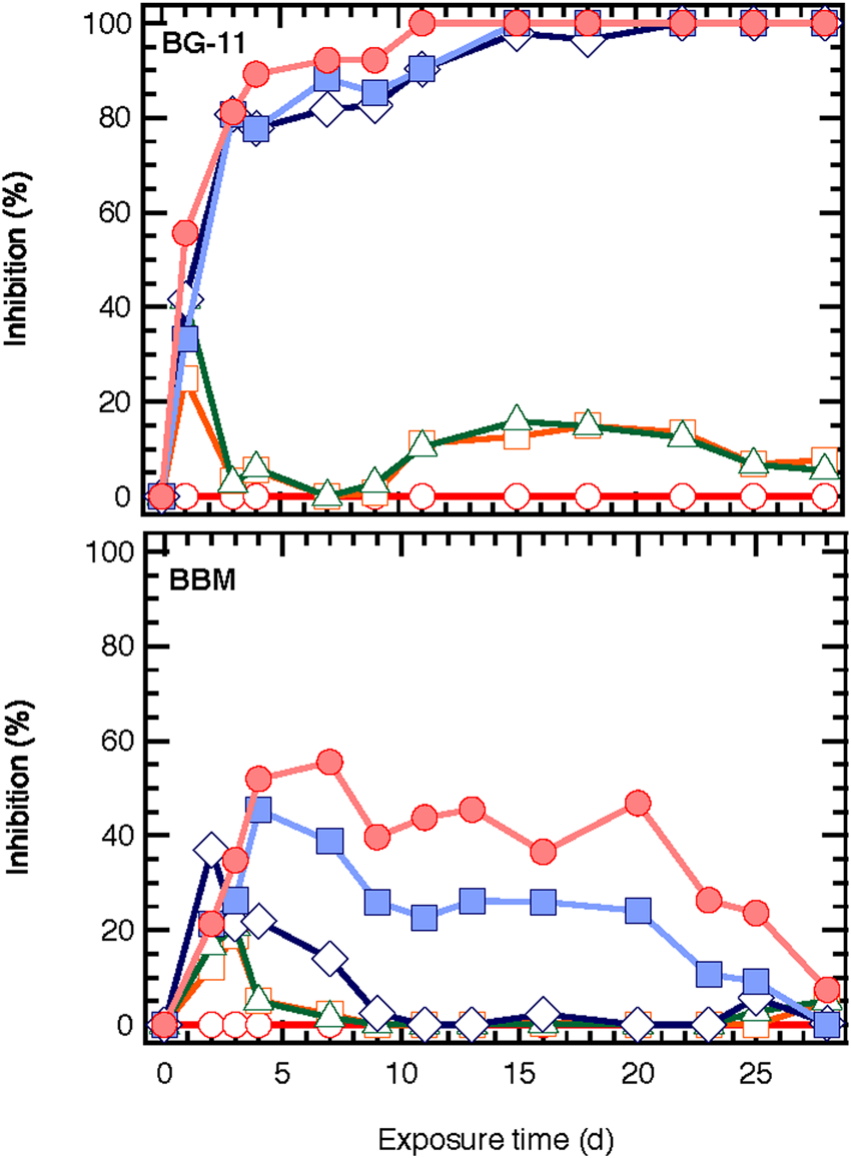
Growth inhibition rate (%I) of ZnO NPs-treated microalgae species (○ 0, □ 0.081, Δ 0.81, ◊ 8.1, ■ 81, ● 810 mg/L ZnO NPs).

Figure 8 illustrates the inhibition of *S. rubescens* exposed to different concentration of ZnO NPs (0.081 to 810 mg/L). ZnO NPs inhibited algal growth even at low concentrations (0.081 mg/L), in both culture media, for short-term exposure (5 days); however inhibitions on the 4^th^ and 7^th^ days were reversed. Long-term toxicity of ZnO NPs to *S. rubescens* demonstrated opposite results, when compared to recent studies of short-term toxicity of ZnO NPs to microalgae^11^. In short-term toxicity assessment of ZnO NPs on *S. rubescens*, lower toxic effects were observed in 1/3N BG-11 compared to BBM cultures. In contrast, in the present long-term toxicity study, ZnO NPs were more toxic in 1/3N BG-11 than in BBM medium cultures. Similar results were reported by Kulacki and Cardinale^22^, who investigated 10 algae species exposed to TiO_2_ NPs for 25 days, in batch conditions and reported that TiO_2_ NPs had little effect on algal growth in contrast to other short-term toxicity studies. The latter confirms our conclusions in a recent study^11^ that the sensitivity of algae to ZnO NPs depends on the exposure time and medium composition. In the following paragraphs an analysis is carried out in order to explain the reasons of the different results between long-term and short-term toxicity assays.

It is worthy to note that significant differences between the two media tested were observed in the cultures exposed to 8.1 mg/L ZnO NPs. A 100% inhibition of *S. rubescens* was observed in the 1/3N BG-11 after 15 days of operation, while in BBM inhibition dropped from 40 to 0% (Fig. 8). The 1/3N BG-11 and BBM media were selected for culturing the *S. rubescens* due to their different Zn^2+^ content, 0.222 mg/L and 8.82 mg/L ZnSO_4_.7H_2_O, or 0.05 and 2.00 mg Zn/L, respectively. At first glance it could be assumed that the presence of higher Zn^+2^ concentration in the ZnO NPs may be more toxic to the algal cultures. However, the increased presence of Zn^2+^ in the BBM medium seems to act positively for algal growth. In other words, when microalgae are cultivated for a long period in high Zn-nutrient medium, they could acquire tolerance in the high Zn environment. Considering that BBM contained 2.00 mg Zn/L, the toxicity to *S. rubescens* on the exposure of low ZnO NPs concentrations, was expected to be lower than to 1/3N BG-11. Admiraal *et al*.^26^, reported that in short-term toxicity tests of zinc to algae, the algae from a strongly polluted environment were only slightly affected by the highest concentrations of zinc tested, in contrast to other algal communities from a cleaner environment.

In the presence of higher ZnO NPs concentrations (81 and 810 mg/L) the inhibition was 100% in case of 1/3N BG-11 medium (Fig. 8). Many studies have shown that high NPs concentration formed aggregates and/or agglomerates, which over the exposure time, were easily settled. This behavior of NPs in the solutions depends on the environmental conditions (pH, ionic composition, organic matter, time, etc.)^6,19,21,22,27,28^. Through the sedimentation of the large aggregates, especially in the highest tested ZnO concentration (810 mg/L), NPs may entrap the algal cells, which are settled and are accounted as toxicity effect. Similar observation has been reported by Ji *et al*.^6^.

Scanning electron microscopy (SEM) images (Fig. S1) confirmed that the addition of ZnO NPs to the algal suspensions triggered the NPs deposition on the algal surface. In this case the most probable mechanism was the direct physical interaction between the algae and NPs^29^. Many other studies have also reported the deposition/wrapping/attachment of NPs onto microalgae cells^6,11,12,15,21,30^. The contact between NPs and algae, could impair the permeability of the cell membrane, which would affect the cell metabolism^29^. Attachment of NPs onto algae could destroy the cell surface architecture, which was also reported by Ji *et al*.^6^, and this could cause leaking of cytoplama^21^. During exposure of microalgae on NPs, self-repair or irreversible damage may occur^29^. This could explain why NPs present different toxicity through exposure time.

At high NPs concentrations aggregates are formed, and the cells are wrapped by them, while at lower concentrations the NPs size decreases^11,18^ and the toxicity increases^18^. Although aggregates could decrease the NPs contact with algae, the creation of aggregations does not mean low toxicity^19^ even though it reduces the NPs dissolution^6^. On the other hand, the small size NPs have the ability to enter into the algal cell^10,17,19,21^, which may cause DNA damage or inhibit photosynthesis by NPs localization in mitochondria^23^. Another impact of algal exposure to NPs, which has been recently reported, is the oxidative stress on the cells^27^ which is caused by the exposure and the reactive oxygen species (ROS) production^6,21,23^. The release of zinc ions from ZnO NPs and the decrease of essential micronutrients caused by ZnO NPs lead to the generation of ROS. Finally, it should be noted that the exact mechanisms for the oxidative effects of ZnO NPs in association with the lack of nutrients, are still unknown. Moreover, in contrast to other types of NPs (TiO_2_, Al_2_O_3_), which present low or zero solubility^30^, ZnO NPs release zinc ions participating to the toxic effects of NPs. Nevertheless, there is a debate for the contribution of zinc ions derived from ZnO NPs to the toxicity on microalgae. Franklin *et al*.^14^, Hartmann *et al*.^21^ and Zhang *et al*.^17^ support that zinc or metal ions play a major role on the toxic effects on microalgae, while Ji *et al*.^6^, believe that they are not the dominant mechanism of toxicity.

The toxic effect of ZnO NPs on algal growth could be estimated more adequately by the half inhibitory concentration (IC_50_) values (Table 1). As it seen much higher values were observed for the cultures with BBM medium. The IC_50_ was decreased with exposure time, especially in cultivation periods where microalgae were stressed by the lack of nutrients. During this period microalgae cells are stressed and are more susceptible to negative effects caused by their exposure to NPs. This is a factor not faced in the short-term toxicity studies, since during the 4-day period the nutrients are in excess. The IC_50_ values of long-term toxicity, should be taken into consideration for specific periods of time, since after the 20^th^ day of culture (with BBM) the cell numbers were decreased even in the absence of NPs. The latter implies that the toxicity results with IC_50_ after that period might be inaccurate. Moreover, nutrient starvation causes death to microalgae cells after some time, which creates debris in the culture. Debris results in the formation of hetero-aggregations, with NPs, which inhibit the toxicity of NPs^6,21^. Therefore, the IC_50_ might be inaccurate after longer periods of exposure, although those periods are closer to real environmental conditions.

**Table 1:** ZnO NP inhibitory concentrations (IC_50_ values) for algal species.

1/3 N BG-11 medium		Bolds Basal Medium	
Exposure time (d)	IC_50_ (mg/L)	Exposure time (days)	IC_50_ (mg/L)
1	28.054	1	>810
3	24.682	3	>810
7	10.166	7	382.805
14	4.797	13	776.74
28	8.393	28	>810

## Conclusions

The results of this work demonstrated that the long-term toxicity tests of ZnO NPs on *S. rubescens* microalgae had different results compared to short-term toxicity tests. Algal growth was significantly affected by the exposure time, the NPs concentration, and mainly the culture medium. Specifically, major differences were observed in the growth rates of *S. rubescens* depending on the composition of the culture medium. Less toxic effects of ZnO NPs were demonstrated with the BBM medium, and algal growth was sustained even at 8.1 mg/L ZnO NPs. The higher zinc content of the BBM medium mightily have affected positively the algal growth due to the adaptation of cells in a high zinc concentration environment. For the determination of algal growth cell number was more realistic than the total suspended solids (TSS) values. The nutrient uptake rate was affected by the presence of ZnO NPs and time of exposure. *S. rubescens* consumed nitrates at different rate in the presence of NPs at 0.081 and 0.81 mg/L in 1/3N BG-11 medium. However, in BBM similar rates were found for ZnO NPs concentration up to 0.81 mg/L. After a period of time nutrient starvation caused the death of microalgae, which was a deterrent to the determination of toxicity with IC_50_ values. Finally, this work revealed the need to study NPs effects on microalgae in real environmental conditions, with different algae species, and for long time exposure.

## Material and Methods

### Nanoparticles

ZnO NPs was purchased from Sigma-Aldrich, USA (catalogue number 544906). The ZnO NPs had particle size smaller than 100 nm and a specific surface area of 15–25 m^2^/g, as reported by the manufacturer. A stock solution of 810 mg ZnO NPs/L in deionized water was prepared and used in experimental studies. The NPs solution was dispersed using an ultrasonic bath before each experiment (TranssonicTI-H-5, Elma Hans Schmidbauer GmbH & Co KG, Gemrany).

### Microalgae

*Scenedesmus rubescens* SAG 5.95 is a freshwater species and was obtained from the bank SAG Culture Collection of the University of Göttingen. *S. rubescens* was selected due to its presence in municipal wastewater and potential use for biofuel production because of its high lipid content.

### Microalgae cultures and ZnO NPs exposure

The experiments were conducted in 2.8 L Erlenmeyer flasks at an initial cell concentration of 10^4^ cells/mL^31^. Microalgae were cultivated in 1/3N BG-11 (BlueGreen-11 enriched with one third times the nitrates concentration) and BBM (Bold’s Basal medium) media recommended for freshwater microalgal species. *S. rubescens* was exposed to ZnO NPs at concentrations from 0.081 to 810 mg/L. This range of ZnO NPs was selected in order to compare short-term toxicity results (previously studied^11^), and considering that ZnO NPs at concentrations lower than 0.081 mg/L did not affect the *S. rubescens* growth^11^. It should be also mentioned that, other studies in the literature^4,6^ report use of relatively higher ZnO NPs concentrations (100 to 1000 mg/L) to investigate the toxicity effects on microalgae. The duration of the experiments was 28 days, and samples were taken at regular time interval in order to evaluate nutrient removal, microalgae growth rate, and lipids content. The experimental system was placed in a walk-in incubator room at a temperature of 20 ± 2 °C. The photosynthetic radiation intensity was 100 μmol m^−2^s^−1^. A continuous air supply of 3 L/min was provided by an air pump (air pump, HP-400, Sunsun, Zhejiang, China), and the air was filtered through a 0.22 μm syringe filter.

### Analytical methods

The effect of NPs on microalgae metabolic reactions was evaluated by the systematic determination of algal biomass, lipid productivity, and nutrient removal. pH, turbidity and temperature were also monitored. Samples were taken every two days to determine nitrates, phosphorus, chl-a, TSS. Microalgal biomass was determined by the measurement of TSS according to Standard Methods^32^. Nutrients (nitrates, nitrites, phosphates and sulphates) were determined by ion chromatography (DX-500, Dionex Coorporation, Sunnyvale, CA, USA). Chl-a was measured via spectrophotometric methods^32^. Briefly, a volume of the culture was filtered through glass fiber filters. The filter was placed in a screw-cap centrifuge tube and 10 mL of 90% acetone was added. The filter was pulverized and shaken in the acetone solution in order to detach algae from the filter and kept at 4 °C in the dark, at least for 2 h. Then the sample was centrifuged and 3 mL of the supernatant was transferred in a cuvette and measured in a spectrophotometer at 750, 665 and 664 nm according to APHA *et al*.^32^.

Lipids were extracted from algal suspensions using a modified Bligh and Dyer method^33^ by gravimetric analysis. Specifically, a measured quantity of dry algal biomass (approximately 100 mg) was homogenized and extracted three times with a chloroform: methanol (2:1) mixture. The biomass was removed by filtration through a filter paper and the extracted lipids transferred quantitatively to a tared Erlenmeyer flask. The procedure was repeated three times. Weight measurements were made by a precision analytical balance (AE200, Mettler Instrumente AG, Zurich, Switzerland). The flask was placed in an oven at 90 °C until all reagents were removed. The flask was allowed to cool to ambient temperature in a desiccator and then was weighed. The weight difference corresponded to intracellular lipids.

### Microscopy monitoring

Scanning electron microscope (SEM/ microscope JEOL 6300, JEOL Ltd.) and optical microscope (model DMLB, Leica Microsystems GmbH) were employed for the direct observation of exposed and non-exposed algae to ZnO NPs, according to Aravantinou *et al*.^11^.

### Data analysis

The specific growth rate (*μ*) was determined according to Aravantinou *et al*.^11^, and the toxicity of ZnO NPs on microalgae was calculated by the half inhibitory concentration values (IC_50_) using Probit Analysis (IBM SPSS software). The growth inhibition rate (I%) was calculated according to the OECD 201 guideline^31^.

1 $${\rm{Inhibition}}\,( \% )=[({{\rm{C}}}_{{\rm{control}}}-{{\rm{C}}}_{{\rm{toxicity}}}){/{\rm{C}}}_{{\rm{control}}}]\,\ast \,100$$

## Electronic supplementary material

Supplementary information
