# Supplementary material for: Long-term toxicity of ZnO nanoparticles to Scenedesmus rubescens cultivated in different media
Source: Sci Rep. 2017 Oct 18;7:13454. doi: 10.1038/s41598-017-13517-7 (PMC5647439; doi:10.1038/s41598-017-13517-7)
Supplement: Supplementary file 1 — Supplementary information [file 41598_2017_13517_MOESM1_ESM.pdf]

Long-term toxicity of ZnO nanoparticles to *Scenedesmus rubescens* cultivated in different media

Andriana F. Aravantinou, Fytoula Andreou and Ioannis D. Manariotis*

Supplementary Information

BG-11		Zoom
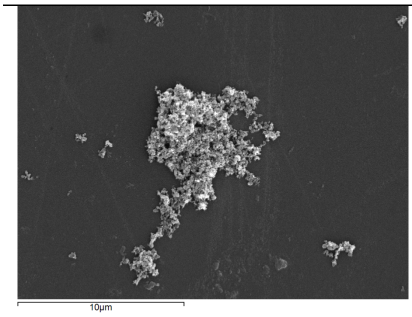	ZnO NPs	x5000
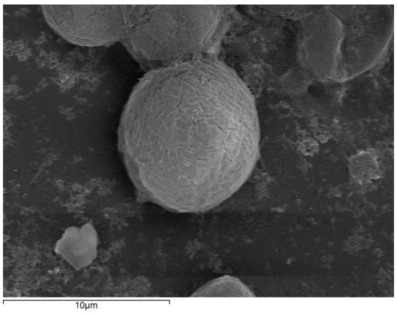	Without ZnO NPs	x5000
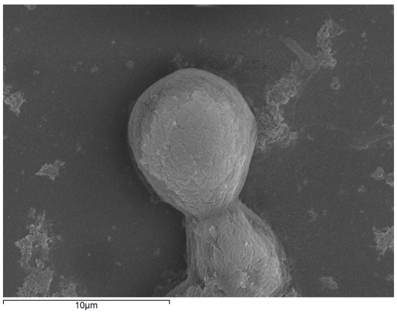	0.81mg/L ZnO NPs	x5000
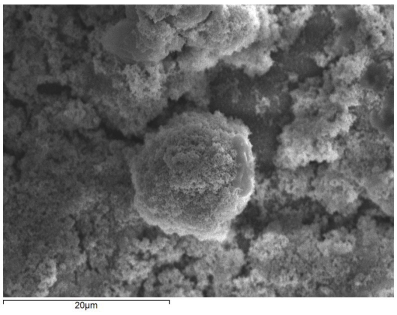	810 mg/L ZnO NPs	x2500

Fig. S1. Representative SEM images of ZnO NPs, and *Scenedesmus rubescens* cultured in ⅓N BG-11 medium, in the absence of ZnO NPs and at 0.81 and 810 mg/L ZnO NPs exposure concentration.
